# Connectivity between ethical sensitivity and empathy ability of nursing students at the initial stage of internship: a bridge network analysis

**DOI:** 10.3389/fpubh.2026.1902943

**Published:** 2026-08-19

**Authors:** Yingxue Tang, Weiwei Xu, Sai Chen, Xiaoping Yang

**Affiliations:** Department of Gastrointestinal Surgery, East Hospital, Zibo Central Hospital, Zibo, Shandong, China

**Keywords:** early stage of internship, empathy ability, ethical sensitivity, network analysis, nursing students

## Abstract

**Objective:**

This study utilized network analysis methods to examine the connection nodes and network correlation characteristics between the ethical sensitivity and empathy skills of nursing students at the beginning of their internship. The interaction mechanism between these two constructs was clarified, thereby providing empirical support for the cultivation of humanistic literacy in clinical nursing education.

**Methods:**

A cross-sectional survey design was adopted from August to September 2025, employing cluster sampling to select 355 nursing students in the early stages of their clinical internship from a tertiary hospital in Zibo City. The Jefferson Scale of Empathy for Healthcare Providers (JSE-HP) and the Nursing Student Ethical Sensitivity Questionnaire (ESQ-NS) were administered to assess the participants. Network analysis was then conducted to construct symptom networks and identify core and bridge symptoms.

**Results:**

The ethical sensitivity score for nursing students at the outset of their internship was 39 (interquartile range: 36, 42), while the average score for empathy ability was 98.19 ± 14.82. Network analysis revealed that items A5, A6, A7, and A13 (representing Items 5, 6, 7, and 13 of the ethical sensitivity scale) and items B1, B6, and B11 (representing Items 1, 6, and 11 of the empathy scale) serve as key bridge connection nodes. These nodes establish a core network path characterized by “emotional perception–ethical decision-making–action implementation.”

**Conclusion:**

Nursing students at the commencement of their internship demonstrate a medium level of ethical sensitivity, while their empathy ability is relatively high. The identified bridge connection nodes play a pivotal role in maintaining the network linkage between ethical sensitivity and empathy skills. Clinical teaching should emphasize ethical dilemmas related to these bridge connections through scenario-based instruction, thereby enhancing the integration of both competencies. This approach will support the development of professional rationality and humanistic care as essential components of nursing students' career literacy.

## 1 Introduction

The clinical internship in hospitals serves as a critical link between academic education and professional practice, representing a vital phase in the career development of nursing students. This period is essential for nursing students to cultivate professional habits and develop their competencies (1). This initial internship period typically lasts less than three months (2), marking the first encounter for nursing students with clinical environments and the complex health needs of patients. During this stage, professional cognition, doctor-patient communication, evolving professional identity, and emotional perception undergo dynamic development. In light of the rapid advancements in medicine and healthcare, alongside the deepening of the bio-psycho-social medical model, the significance of nursing practice has become increasingly evident. The role of nurses has evolved from mere disease management to holistic emotional care; thus, ethical sensitivity and empathy significantly impact patients‘ recovery experiences (3, 4). Nevertheless, nursing students' sensitivity in emotional perception—including aspects such as professional identity, self-concept, moral ethics, and empathy—remains significantly lower than that of practicing nurses.

Ethical sensitivity allows nursing students to recognize that all clinical practices and behaviors must align with ethical principles. A deficiency in ethical sensitivity can lead to non-standard nursing practices (5). Consequently, ethical sensitivity represents a primary prerequisite for nursing students, facilitating ethical decision-making and behavior implementation, which in turn aids in accurately identifying patients‘ ethical needs and mitigating ethical risks in complex clinical scenarios (6). Empathy ability refers to the emotional capacity of nursing students to adopt different perspectives, consider situations from the patients' viewpoints, and experience emotions that illuminate core demands. This capability forms the foundation for establishing trustworthy communication between nurses and patients and critically influences the focus and efficacy of clinical nursing work (7). From a psychological standpoint, empathy is viewed as an emotional response generated by an individual's comprehension of another person's emotional state or circumstances (8).

Kohlberg, building on Piaget's theory of moral cognitive development, proposed a three-level, six-stage model of moral development (9). Initially centered on children and adolescents, this theoretical framework was subsequently expanded by Rest, a student of Kohlberg, to broaden its applicability (10, 11). According to Kohlberg's theory, moral emotions serve as the essential motivational precursors for the emergence of moral cognition (12). In his foundational study, Kohlberg emphasized that mere acquisition of rules is insufficient to foster active moral awareness. Instead, the empathetic experience of witnessing others' suffering acts as a catalyst for moral reasoning, propelling individuals from pre-conventional self-interest to more advanced moral cognition (9). Individuals exhibiting high levels of emotional empathy are adept at perceiving the underlying emotions and situational challenges faced by service recipients, including pain, helplessness, and rights violations (13). They are capable of transcending cognitive inertia and emotional numbness in professional contexts (14), actively addressing ethical contradictions that may be easily overlooked. This emotional engagement not only awakens ethical awareness from an affective perspective (12) but also enhances the sensitivity to ethical issues, providing the emotional basis for the development of ethical sensitivity (15). Numerous empirical studies within the nursing field (16–18) have substantiated that emotional empathy is significantly positively correlated with ethical sensitivity. Nurses possessing strong emotional empathy are better equipped to recognize the implicit ethical needs of patients and deliver more compassionate care services.

Nursing students' empathy can offer essential emotional support for the development of ethical sensitivity, while enhanced ethical sensitivity facilitates the translation of empathetic feelings into ethically compliant nursing practices (7). Existing studies have predominantly investigated the independent status (19, 20), influencing factors, and cultivation strategies of these two constructs; however, research focusing on their internal correlation and interaction remains limited.

Bridge connection network analysis, a methodological approach within psychological network analysis (Psychological Network Analysis, PNA) (21, 22), centers on the concept of bridge connection nodes. In a network comprising two independent variable systems, bridge connection nodes are those elements that belong to both systems, facilitating cross-system information transmission and serving as intermediaries. The pathways that link the two sets of variables are termed bridge connection paths. This type of network analysis quantifies direct associations between variables, incorporating all elements into a comprehensive network for a holistic analysis of their interactions (23). Both empathy and ethical sensitivity are multi-dimensional latent variables; therefore, the network analysis model can illustrate the degree of mutual influence among the items within these variables, extending beyond the mere total effect reflected by regression or mediation analyses. Bridge connection network analysis provides a more intuitive presentation of the interconnections and transmission pathways among each item, efficiently highlighting core nodes that illustrate the influence of empathy on ethical sensitivity (24). Interventions targeting these bridge connection nodes can trigger chain reactions throughout the network, resulting in more rapid and effective improvements to the desired outcomes (25). This study focuses on nursing students in their initial internship period as research subjects and employs bridge network analysis to explore the mechanisms underlying the association between ethical sensitivity and empathy. The findings are intended to inform the identification of key priorities and weaknesses in the development of professional literacy among early-internship nursing students, ultimately optimizing nursing education practice programs and assisting students in their transition from the role of students to that of practicing nurses.

## 2 Methods

### 2.1 Study design and study participants

From August to September 2025, a cluster sampling method was utilized to recruit all junior college and undergraduate nursing interns with 1 to 2 months of internship experience at a Grade 3 Class A hospital in Zibo City. The inclusion criteria were as follows: (1) Full-time junior college or undergraduate nursing students who had completed on-campus theoretical courses and had commenced the clinical internship phase; (2) Duration of clinical internship less than 3 months; (3) Absence of long-term leave ( ≤ 1 week) or interruptions during the internship period to ensure a continuous early internship status; (4) Proficient reading, writing, and comprehension skills in Chinese; (5) Voluntary participation in the study.

Exclusion criteria included: (1) Individuals with severe mental illnesses (e.g., depression, anxiety disorders), cognitive impairments, or language barriers that would prevent them from completing the questionnaire assessment appropriately; (2) Individuals who withdrew from the questionnaire survey midway; (3) Individuals previously involved in systematic intervention studies on nursing students' ethical sensitivity or empathy (e.g., training or psychological counseling) that could potentially affect the measurement of indicators in their natural state.

This study employed a cross-sectional survey design. The sample size was estimated using the formula for Pearson correlation analysis of two continuous variables (26). The formula is as follows: *n* = *r*^2^ (*Z*_*α*_/_2_+ *Z*_*β*_)^2^ × (1–*r*^2^)^2^, where n represents the base sample size, α = 0.05 is the two-sided test level, (*Z*_*α*/2_ = 1.96); the test power 1 - *β* = 0.8, *Z*_*β*_ = 0.84; and *r* denotes the expected correlation coefficient between the two variables. Based on findings from similar cross-sectional studies in the field of nursing in China, the overall correlation coefficient between nurses or nursing students' empathy and ethical sensitivity is generally observed to be within the range of 0.3–0.4 (18, 27, 28). When calculated with *r* = 0.3 (indicating a weak correlation) and *r* = 0.4 (indicating a moderate correlation), the base sample sizes are determined to be 72 cases and 35 cases, respectively. An increase of 20% in sample size was recommended to compensate for potential losses due to invalid questionnaires and missing data. After adjustment, the minimum required sample size was established at 87 cases.

In this study, the cluster sampling method facilitated a questionnaire survey among all students undertaking internships at the tertiary hospital in Zibo City. A total of 392 questionnaires were distributed, yielding 355 valid responses, resulting in an effective recovery rate of 90.6%.

### 2.2 Data collection

#### 2.2.1 General information

The general situation questionnaire, developed by the researchers, collected demographic information such as gender, age, place of residence, only-child status, reasons for selecting the nursing profession, and parental marital status.

#### 2.2.2 Ethical Sensitivity Questionnaire for Nursing Students (ESQ-NS)

The Ethical Sensitivity Questionnaire for Nursing Students (ESQ-NS) was employed to measure the ethical sensitivity of nursing students at the commencement of their internships. This questionnaire was originally developed by Muramatsu et al. (29) and subsequently translated into Chinese by Na et al. (15). It comprises 13 items spanning three dimensions: respect for individuals, fair distribution, and protection of patient privacy. A Likert four-point scale is utilized, with total scores ranging from 13 to 42. The results are not categorized into distinct grades; higher scores indicate greater levels of ethical sensitivity among nursing students. Psychometric validation of this scale has been conducted among clinical nurses in the Chongqing region (15), yielding a Cronbach's α coefficient of 0.865. In this study, the Cronbach's α coefficient for the scale was calculated at 0.797.

#### 2.2.3 Jefferson scale of empathy-health professionals (JSE-HP)

The Jefferson Scale of Empathy (JSE), initially established in 2001 by Dr. Mohammadreza Hojat and his team (30) and localized for the Chinese nursing population by Xiuqin et al. (31), yielded a Cronbach's α coefficient of 0.750, demonstrating good reliability and validity. This scale consists of 20 items divided into three dimensions: perspective-taking, emotional care, and empathy-switching. A Likert seven-point rating system is employed, with total scores ranging from 20 to 140. Similar to the ESQ-NS, the total score is not categorized into specific grades; higher scores reflect elevated levels of empathy among nursing interns. In this study, the Cronbach's α coefficient for this scale was found to be 0.818.

### 2.3 Quality control

To ensure the accuracy and reliability of the investigation results, the researchers received training on the research process. All participants completed the questionnaires in a unified and private environment, with measures taken to protect their privacy. An online questionnaire method was utilized, with questionnaires collected immediately after completion, ensuring that each individual could submit only one questionnaire. Invalid responses, due to improper completion, were excluded from the analysis. The entire investigation adhered to ethical guidelines and standardized procedures, with meticulous attention to scientific rigor, fairness, and consistency in every operational step.

### 2.4 Statistical analysis

Descriptive and univariate analyses were performed using SPSS 25.0 (IBM Corporation, Armonk, New York, USA). For measurement data that adhered to a normal distribution, results were presented as mean ± standard deviation (x ± s). Univariate analyses utilized *t*-tests or analysis of variance to assess differences. For measurement data that did not conform to a normal distribution, the median (with first and third percentiles) was reported, denoted as M (P25, P75). The Mann–Whitney *U* test or Kruskal–Wallis *H* test was employed for univariate analyses, with a significance level established at α = 0.05.

In this study, RStudio software, along with the bootnet and qgraph packages, facilitated the construction of the network model and the generation of visual diagrams. Each node in the visualization represented an individual item from the scale, while the edges connecting the nodes illustrated the correlation relationships among these items. The thickness of the edges indicated the strength of correlation, with thicker lines reflecting a higher degree of association. The study calculated three fundamental network centrality indicators: strength centrality (SC), closeness centrality (CC), and betweenness centrality (BC). SC quantifies the significance of a node within the overall network by summing the weights of all edges associated with it. CC is defined as the reciprocal of the total shortest path distance from a node to all other nodes within the network. BC represents the frequency with which a node lies on the shortest path connecting any two other nodes (32).

For bridge centrality evaluation, this study selected bridge strength, bridge proximity centrality, and bridge mediating centrality as indicators. Bridge strength refers to the sum of the direct correlation weights of a specific item with all items in another variable system, reflecting the transmission effect of this item on that system. Bridge proximity centrality represents the average distance from the bridge item to all nodes in the alternative system, indicating the hub position of this item within the cross-system network. Bridge mediating centrality measures the number of shortest paths that any pair of items from the two variable systems traverse through this bridge item, assessing its cross-system mediating transmission capability (21).

Utilizing the bootnet program package, the 95% confidence intervals for the weights and centrality indicators of each associated edge were estimated through the Bootstrap method. The accuracy of the network estimation results was evaluated, and the correlation stability coefficient (CS coefficient) was calculated to determine the stability of the indicators.

## 3 Results

### 3.1 Analysis of the differences in ethical sensitivity and empathy ability among nursing students with different demographic characteristics

A total of 355 nursing students participated in this study, with a median age of 21 years (interquartile range: 20, 21). These students recorded an ethical sensitivity score of 39 points (interquartile range: 36, 42) and an empathy ability score of 98.19 ± 14.82 points. Statistically significant effects on the ethical sensitivity score were observed related to the reason for choosing the nursing major (*P* = 0.006). Gender also significantly influenced the empathy ability of the nursing students (*P* = 0.049), with female nursing students scoring higher (99.03 ± 14.56) than their male counterparts (95.33 ± 15.44). Furthermore, the reason for selecting the nursing major significantly affected the empathy ability score (*P* = 0.004), with nursing students who voluntarily chose the nursing profession exhibiting the highest empathy levels. The marital status of parents significantly impacted nursing students' empathy ability (*P* = 0.041), with those from stable parent relationships achieving the highest empathy scores. A positive correlation between age and empathy ability was found (*Rs* = 0.174, *P* = 0.001). Additional general demographic data for the nursing students can be found in Table 1.

**Table 1:** General demographic characteristics (*N* = 355).

Demographic characteristics	Subgroup categories	*N*(%)	ESQ-NS	JSE-HP
Gender	Male	80 (22.5)	39 (36, 43)	95.33 ± 15.44
	Female	275 (77.5)	38 (36, 41)	99.03 ± 14.56
	*Z*		−1.620	−1.974
	*P*		0.105	0.049
Current residence	Cities and towns	153 (43.1)	39 (36, 41)	97.00 ± 15.32
	Countryside	202 (56.9)	38 (36, 42)	99.09 ± 14.40
	*Z*		−0.550	−1.32
	*P*		0.583	0.188
Only child	Yes	75 (21.1)	39 (36, 42)	97.44 ± 15.86
	No	280 (78.9)	38.5 (36, 42)	98.39 ± 14.55
	*Z*		−0.678	−0.494
	*P*		0.498	0.622
The reasons for choosing the nursing profession	Volunteer	162 (45.6)	39 (36, 43)	101.27 ± 14.12^a^
	The wishes of parents or family members	140 (39.4)	38 (35, 40)	95.34 ± 14.52^a^
	Comply with the allocation	25 (7.0)	40 (37, 42)	98.36 ± 17.88
	Else	28 (7.9)	38 (35, 39)	94.50 ± 14.41^a^
	*Z*		12.577	8.246
	*P*		0.006	0.004
Parental relationship status	Good	332 (93.5)	39 (36, 42)	98.51 ± 14.63 ^a^
	Divorced	13 (3.7)	39 (36.5,43)	98.38 ± 17.71
	Widowed or otherwise	10(2.8)	37 (33.25,40)	87.40 ± 14.69 ^a^
	*Z*		1.894	4.192
	*P*		0.388	0.041
Hold the position of a student council officer	Yes	131 (36.9)	39 (36, 42)	97.97 ± 14.92
	No	224 (63.1)	39 (35, 41)	98.32 ± 14.79
	*Z*		−0.451	−0.216
	*P*		0.652	0.829
Age		21 (20, 21)^b^		
	*R*		0.032	0.174
	*P*		0.542	0.001

ESQ-NS, The Ethical Sensitivity Questionnaire for Nursing Students; JSE-HP, The Jefferson scale of empathy-health professionals; ^a^For post-test verification, it is statistically significant; ^b^Non-normal distribution, represented by quartiles; *Z* is the normal approximation statistic of the Mann-Whitney U rank sum test after correction for the same rank order; r: Spearman's rank correlation coefficient

### 3.2 Multivariate regression analysis of nursing students' ethical sensitivity

A hierarchical multiple linear regression analysis was performed to explore the independent predictive effect of empathy on the ethical sensitivity of nursing students. The total score of ethical sensitivity served as the dependent variable in a two-level regression model. The first level included seven demographic confounding variables: gender, age, place of residence, only-child status, reason for choosing the nursing major, parental marital status, and whether the individual served as a student leader. The second level introduced the total empathy score as the core predictive variable. Collinearity diagnostics indicated that the variance inflation factor (VIF) values for all independent variables were below 1.2, suggesting no concerns with multicollinearity. Model 1, which comprised only demographic variables, did not yield statistical significance (*F* = 1.339, *P* = 0.231), with an adjusted *R*^2^ of 0.007. The reason for choosing a career was found to weakly predict ethical sensitivity (*P* = 0.021). In Model 2, after incorporating the total empathy score, the overall model achieved significance (*F* = 5.574, *P* < 0.001), resulting in an adjusted *R*^2^ of 0.094. Controlling for all demographic confounding factors, the total empathy score emerged as a positive and independent predictor of ethical sensitivity (*B* = 0.108, β = 0.309, *t* = 5.859, *P* < 0.001). This suggests that higher empathy levels correspond to greater ethical sensitivity among nursing students. Among the demographic variables, only gender exhibited a weak predictive effect (*P* = 0.038), while other demographic characteristics did not demonstrate independent predictive effects (see Table 2).

**Table 2:** Multivariate regression analysis of medical students' ethical sensitivity.

Predictors	β	*t*	*Sig*	*VIF*	β	*t*	*Sig*	*VIF*
	Model 1				Model 2			
Constant		6.500	0.000			5.504	0.000	
Gender	0.080	1.429	0.154	1.126	0.112	2.082	0.038	1.138
Age	0.051	0.952	0.342	1.028	−0.003	−0.058	0.954	1.062
Current residence	0.027	0.478	0.633	1.118	0.039	0.722	0.471	1.120
Only child	0.000	0.003	0.998	1.216	−0.007	−0.130	0.896	1.217
The reasons for choosing the nursing profession	−0.124	−2.312	0.021	1.025	−0.076	−1.473	0.142	1.051
Parental relationship status	−0.051	−0.950	0.343	1.026	−0.021	−0.413	0.680	1.036
Hold the position of a student council officer	−0.040	−0.733	0.464	1.066	−0.034	−0.658	0.511	1.067
JSE-HP	—				0.108	5.859	0.000	1.083
*R^2^*	0.026				0.114			
*Adj. R^2^*	0.007				0.094			
*F*	1.339				5.574			

*β*, standardized regression coefficient; t, t-statistic; Sig, significance P-value; VIF, variance inflation factor; Model 1 only included demographic variables; Model 2 added the total score of Jefferson Scale of Empathy-Health Professionals (JSE-HP) on the basis of Model 1; *R*^2^, coefficient of determination; Adj. *R*^2^, adjusted *R* square; *F, F*-statistic of regression model; JSE-HP, The Jefferson scale of empathy-health professionals.

### 3.3 Analysis model of nursing students' ethical sensitivity and empathy ability based on bridge connection network and centrality indicators

**Figure 1:**
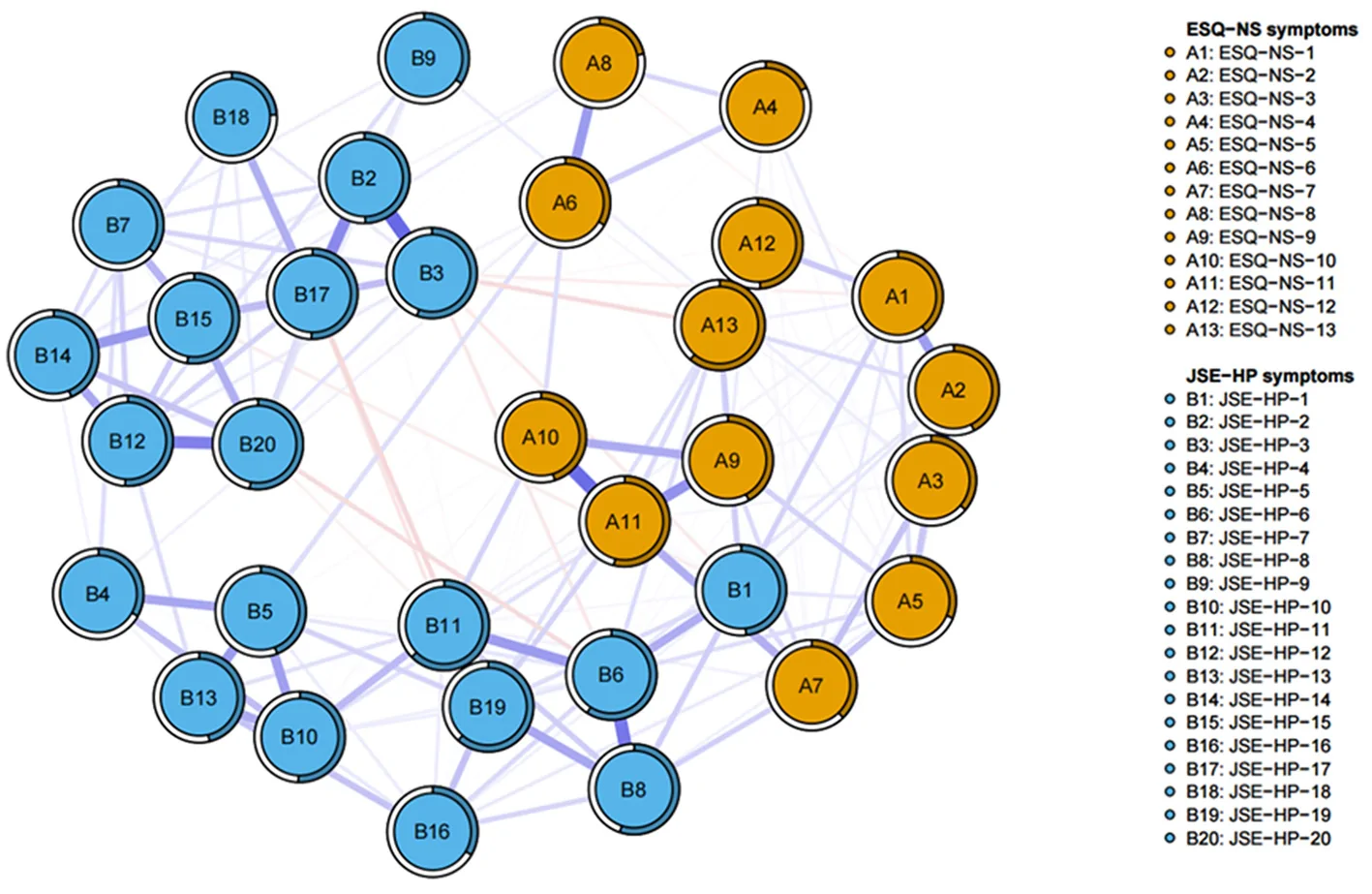
Network structure of nursing students ethical sensitivity and empathy ability. ESQ-NS, The Ethical Sensitivity Questionnaire for Nursing Students; JSE-HP, The Jefferson scale of empathy-health professionals; See Appendix 1 for detailed item names. Orange nodes represent ethical sensitivity, and blue nodes represent empathy ability.

The network analysis model examining ethical sensitivity and empathy ability among nursing students was constructed using R version 4.5.2 (The R Foundation for Statistical Computing, Vienna, Austria) and RStudio (Posit Software, PBC, Boston, Massachusetts, USA). The network comprised 33 nodes, showcasing relatively tight connections among items. A total of 167 non-zero edges out of a possible 528 were identified, resulting in a network connection density of 0.023. According to the structural model diagram from the network analysis (Figure 1), among the 13 indicators of ethical sensitivity in nursing students, the correlation weight between A12 (Reporting the patient's condition to the responsible nurse in the ward) and A13 (Reporting the detailed situation of patient care to the head nurse of the ward) was the highest (*Rs* = 0.782, *P* < 0.001). Among the 20 indicators assessing empathy ability, the strongest correlation was found between B6 (In my relationship with the patient, understanding their body language and verbal communication is equally important) and B8 (I will pay attention to the patient's displayed body language and non-verbal cues to understand what the patient is thinking) (*Rs* = 0.695, *P* < 0.001). The strongest connection between ethical sensitivity and empathy ability was identified between A13 (Reporting the detailed situation of patient care to the head nurse of the ward) and B1 (In my relationship with the patient, understanding the emotional state of the patient and their family is an important factor) (*Rs* = 0.496, *P* < 0.001).

**Figure 2:**
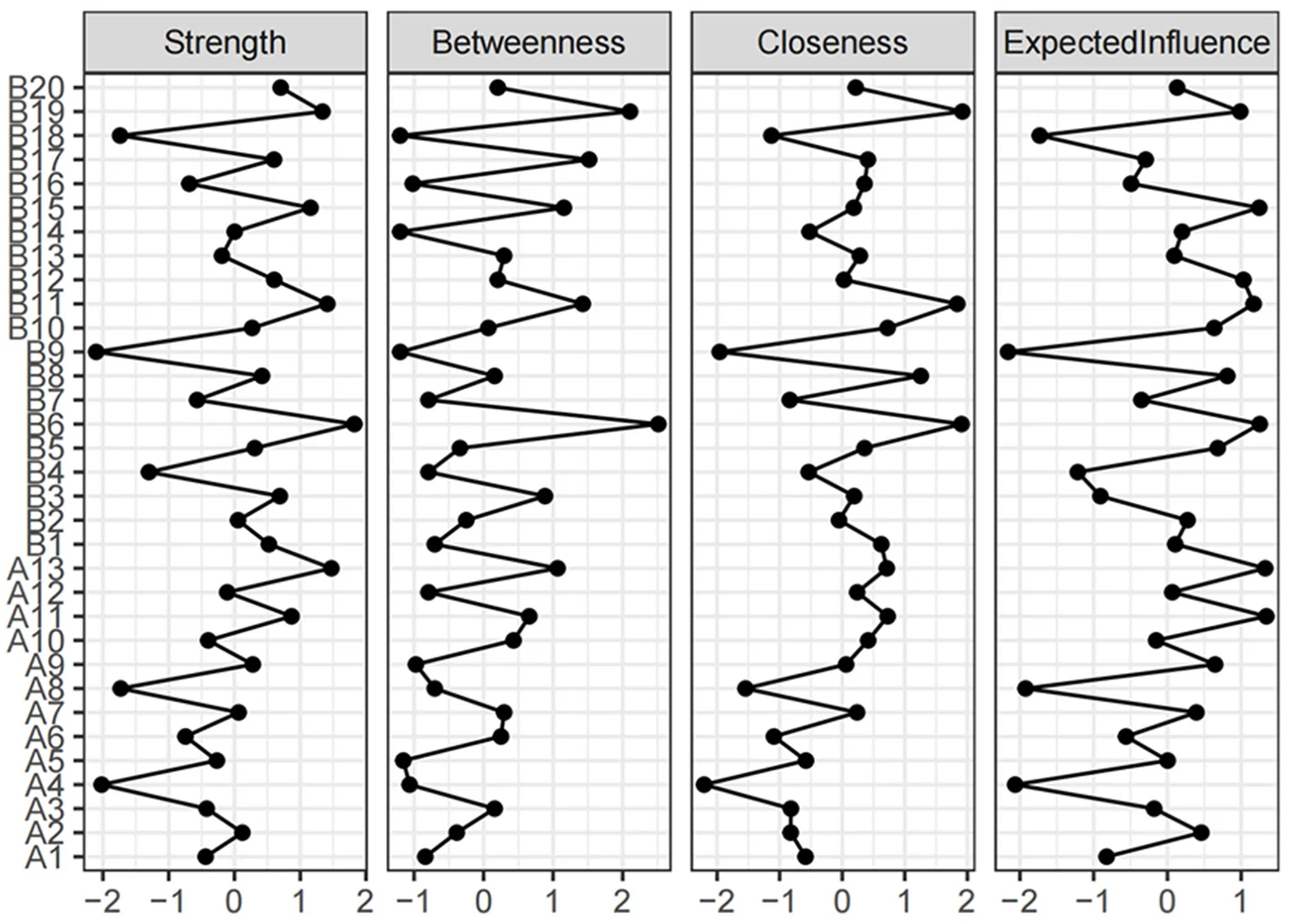
Standardized estimated value of the central indicators of ethical sensitivity and empathy ability of novice nursing students. ESQ-NS, The Ethical Sensitivity Questionnaire for Nursing Students; JSE-HP, The Jefferson scale of empathy-health professionals; See Appendix 1 for detailed item names. Orange nodes represent ethical sensitivity, and blue nodes represent empathy ability.

Standardized estimated values for the model's centrality indicators were illustrated in Figure 2. SC, which measures the direct influence of a node, identified the top three nodes as B6, A13, and B11 (In order to provide better nursing services, I will try to consider the problem from the patient's perspective). BC, reflecting a node's hub role in information transmission, highlighted B6, B19 (When caring for patients, I will try to think from the patient's perspective), and B17 (For me, thinking from the patient's perspective is a very difficult thing). CC, which indicates the efficiency of information dissemination within the network, ranked the top three nodes as B19, B6, and B11. Expected influence, which assesses a node's potential impact on information dissemination, placed A11 (To adapt to the eating speed of patients with swallowing difficulties and provide assistance, including at least 1 h of uninterrupted supervision), B13 (I empathize with the patients, and they will feel that the treatment is effective), and B6 at the top three positions.

**Figure 3:**
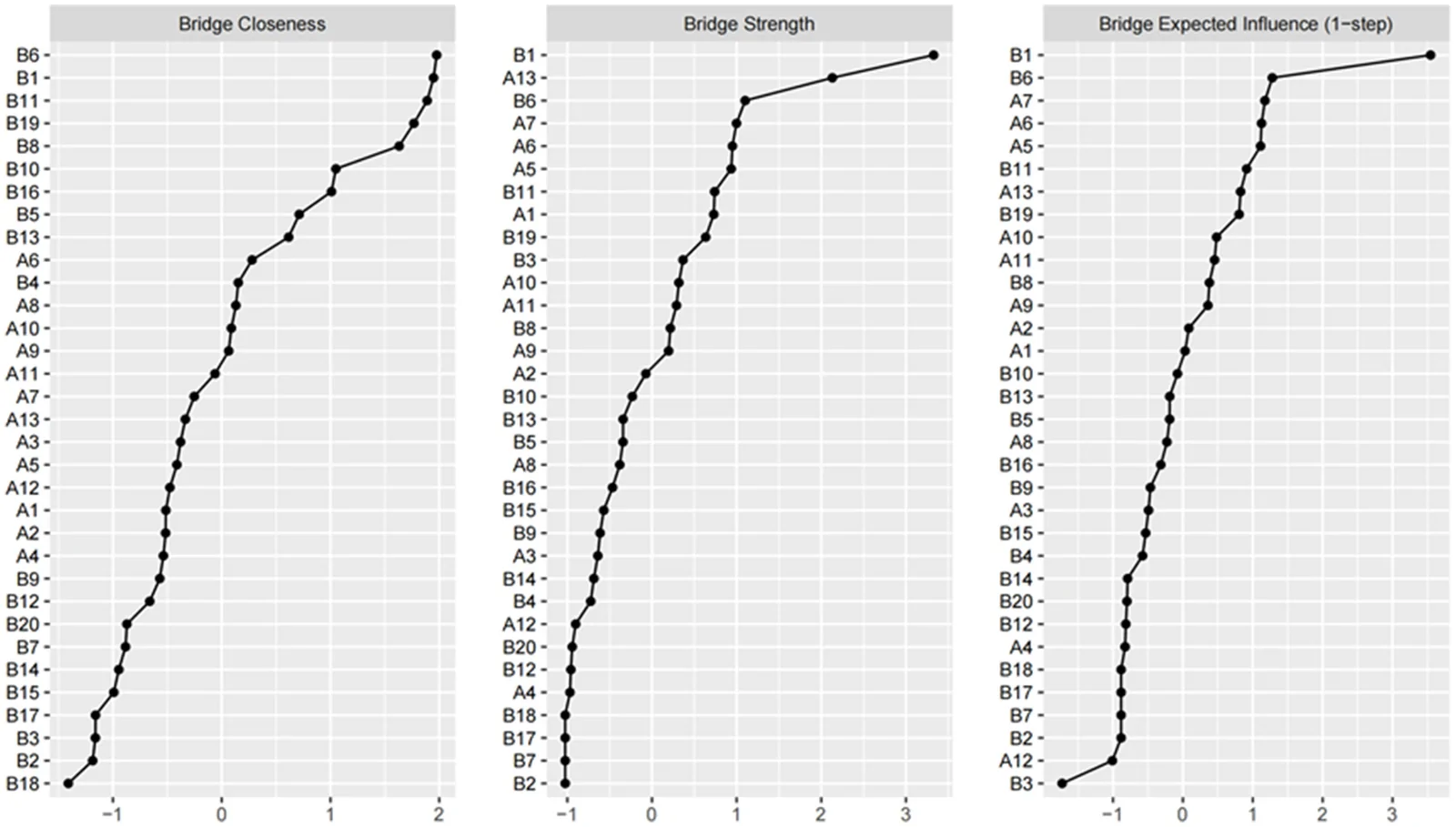
Network analysis of ethical sensitivity and empathy ability of nursing students (Bridge Centrality Index). ESQ-NS, The Ethical Sensitivity Questionnaire for Nursing Students; JSE-HP, The Jefferson scale of empathy-health professionals; See Appendix 1 for detailed item names. Orange nodes represent ethical sensitivity, and blue nodes represent empathy ability.

**Figure 4:**
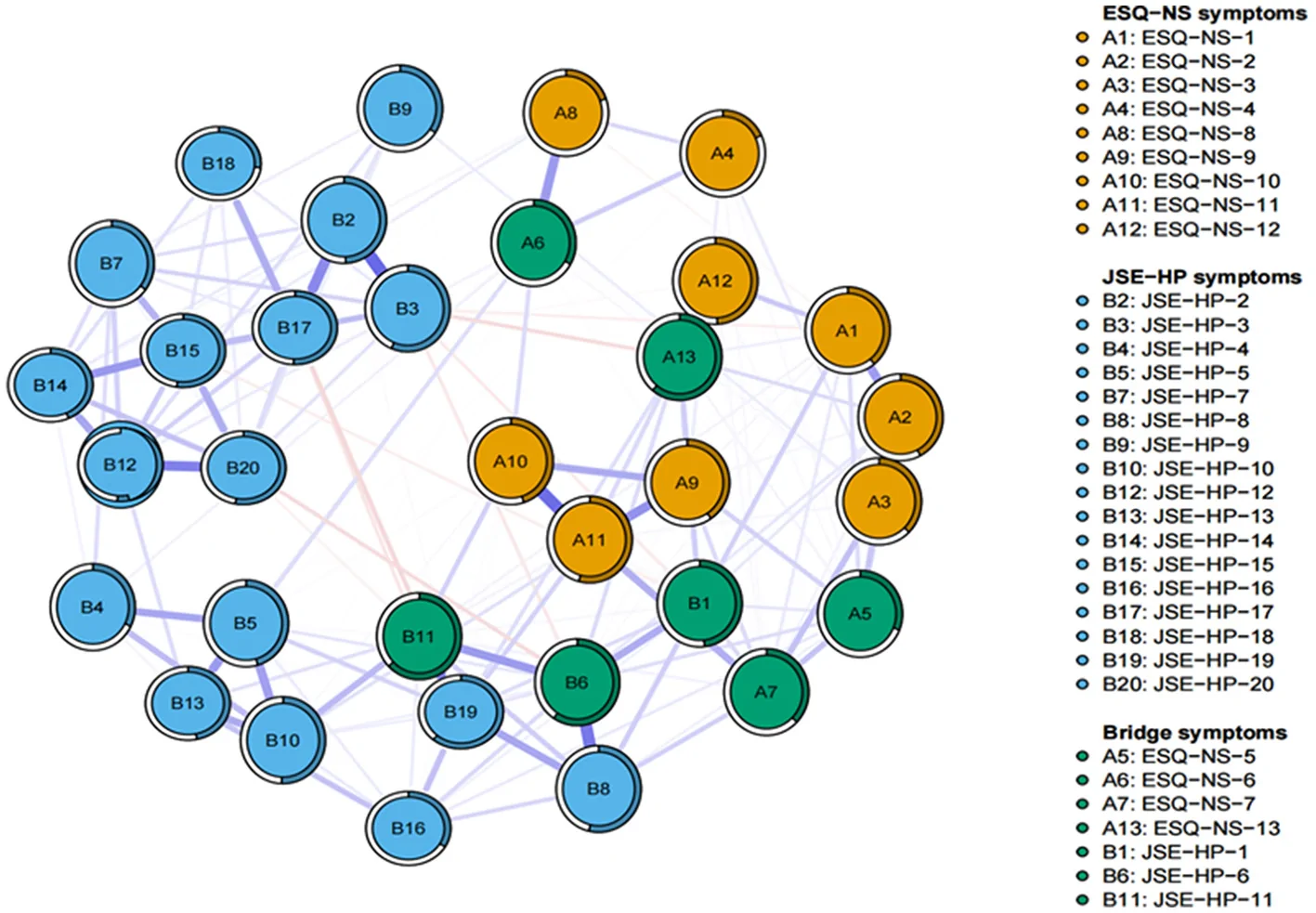
The bridge connection network structure of medical students ethical sensitivity and empathy ability in the early intership period. Orange node represent ethical sensitivity, blue node represent empathy ability, and green nodes represent bridge connection nodes. ESQ-NS, The Ethical Sensitivity Questionnaire for Nursing Students; JSE-HP, The Jefferson scale of empathy-health professionals; See Appendix 1 for detailed item names. Orange nodes represent ethical sensitivity, and blue nodes represent empathy ability.

The node with the highest centrality in the bridge connection network is identified as B6 (refer to Figure 3). The bridge connection nodes relevant to the ethical sensitivity and empathy ability of nursing students in their initial practical experience include B1, B6, B11, A5 (If sensor pads are used, they should be placed beside the beds of patients with a high risk of falls), A6 (Dementia patients who can sit in wheelchairs and fasten seat belts can stay at the nurse station), A7 (Male patients refuse to be cared for during bathing, but due to their condition and safety requirements, it should be done after persuading the patients to agree), and A13 (refer to Figure 4).

### 3.4 Network analysis stability test of nursing students' ethical sensitivity and empathy ability

**Figure 5:**
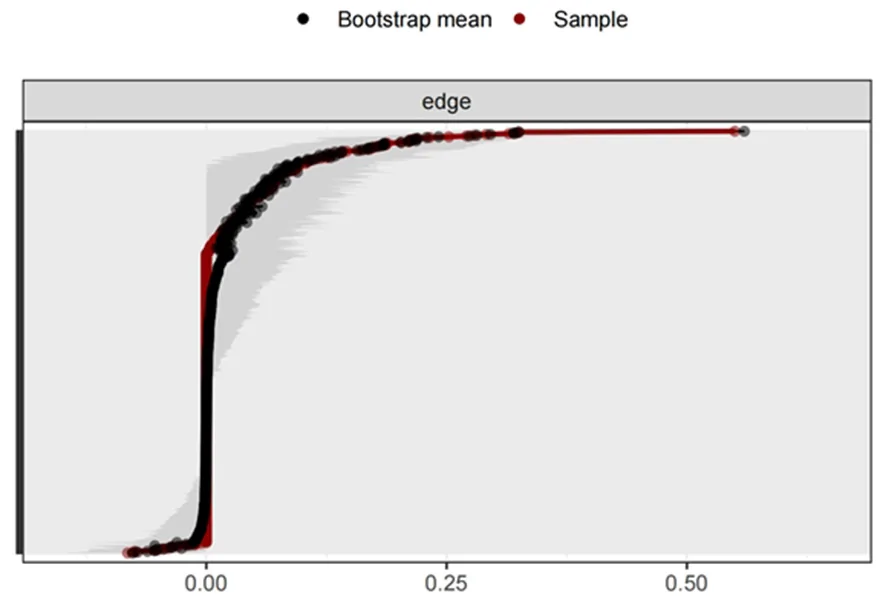
Estimation of edge weight accuracy.

The network's average marginal weights, represented by black lines, were derived through the self-assessment method. The red lines depict the marginal weights specific to the sample in this study, while the gray area indicates the 95% confidence interval. The relatively narrow 95% confidence interval of the marginal weights suggests a high level of accuracy in the assessment (Figure 5).

**Figure 6:**
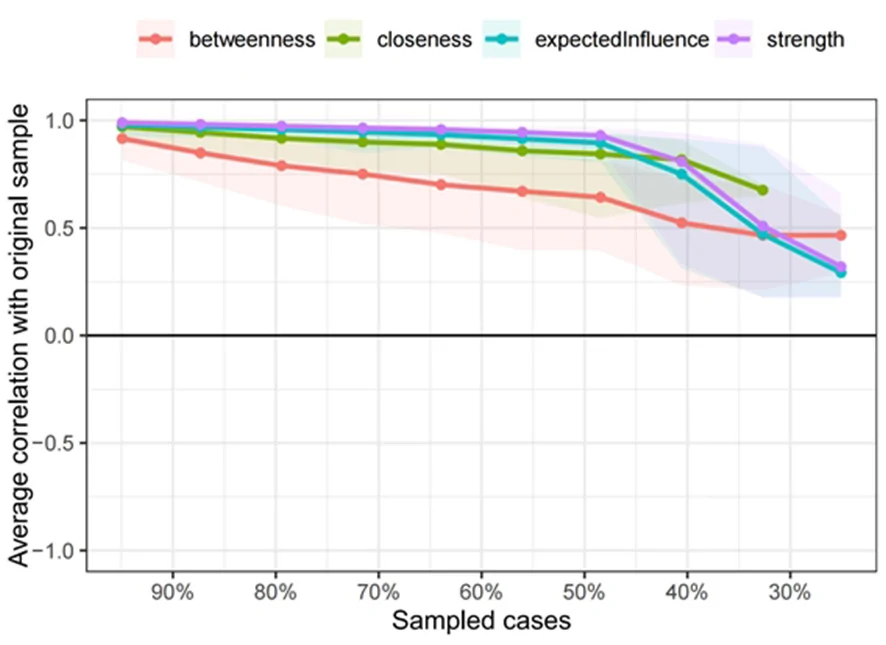
Central index stability test.

The analysis model of the bridge connection network elucidating the relationship between ethical sensitivity and empathy ability among nursing students during their initial internship indicated the following centrality measures: SC at 0.515, BC at 0.327, CC at 0.361, and expected influence at 0.515 (Figure 6). Values exceeding 0.25 for these centrality metrics imply that the stability of the network model is acceptable, while scores surpassing 0.50 indicate a robust stability level (33).

## 4 Discussion

### 4.1 Analysis of the current status of ethical sensitivity and empathy skills of nursing students in the early stage of internship

The results of this study demonstrate that the ethical sensitivity score of nursing students was 39 (34, 35) points. In comparison to the findings of Hongyuan et al. (6), the average scores for the two groups exhibited minimal differences. However, an analysis of the dispersion trend indicated that the scores in this study were more concentrated. This observation may be attributed to the participants being nursing students in the initial stages of their internship, resulting in relatively limited clinical experience and consequently a higher degree of homogeneity. The ethical sensitivity score in this study was lower than that reported by Niu et al. (36), a difference that could be linked to the fact that 90% of the nursing students involved in this study had received training in nursing ethics. The empathy score of the nursing students was recorded at 98.19 ± 14.82 points, which surpassed the scores of nursing students in the studies by Min et al. (37) and Huimin et al. (34), both of whom had completed over 6 months of internship. Initially, nursing students enter practice with high levels of professional enthusiasm. However, as the internship progresses, they gradually adapt to the clinical environment. Increased clinical workloads may lead to experiences of occupational burnout and emotional exhaustion (38), resulting in a potential decline in empathy ability, understood as a form of emotional labor. Furthermore, when nursing students confront patients' suffering and death for the first time, their emotional responses typically exhibit significant fluctuations. Continuous exposure to such emotional circumstances may result in emotional adaptation or desensitization, thereby affecting their empathy ability over time (39). It is recommended that the development of ethical and empathetic skills among nursing students occur throughout the entire internship period. A phased training plan should be designed after assessing the specific contexts and needs of the nursing students at different stages of their internship.

### 4.2 Analysis of the bridge connection nodes between ethical sensitivity and empathy ability of nursing students at the initial stage of internship

This study identified the connection nodes between ethical sensitivity and empathy ability among nursing students at the initial internship stage as A5, A6, A7, A13, B1, B6, and B11. Further details regarding these nodes can be found in Appendix 1, which provides a brief overview of their contents.

*The stimulation of nursing students' high ethical sensitivity behavior is inseparable from the driving force of empathy ability*.

Results indicated that B6 (In my relationship with the patients, understanding their body language and verbal communication are equally important) exhibited the highest bridge centrality. This suggests that B6 plays a central role in the interaction between ethical sensitivity and empathy ability among nursing students.

As a single-region cross-sectional study, the research primarily involved participants from Shandong, China. The local context, characterized by its unique doctor-patient communication atmosphere, public health concepts, and nursing ethics education, reflects distinctive features of the North China region. Research has demonstrated that cross-regional cultural differences can influence individuals' verbal and non-verbal emotional signals (40), which may affect the intensity of the core bridge items in this study. Consequently, further multi-center and cross-regional studies with larger samples are necessary to verify the generalizability of these conclusions. In daily nurse-patient communication, while verbal communication typically dominates, the significance of patients' body language should not be overlooked. Studies indicate that when nurses accurately interpret patients' body language and respond appropriately, this enhances the establishment of a positive nurse-patient relationship during the early stages of treatment, fosters intimacy, and stimulates nurses' empathetic responses toward patients (41). Additionally, Sharifnia et al. (42) noted that empathy directly influences nurses' ethical sensitivity and behavior. When nursing students empathize with a patient's underlying fear and discomfort, as highlighted in B1 (Paying attention to the emotional state of the patient and their family), their ethical sensitivity becomes significantly activated (35). A higher level of empathy correlates with nursing students' propensity to make prudent judgments and execute ethical care behaviors while balancing “respecting the patient's autonomy” and “ensuring the patient's safety” (43). Thus, incorporating training on interpreting body language communication into the early education of nursing students is recommended. Such training could include activities like watching silent videos and engaging in role-playing exercises. By enhancing students' empathy and thereby influencing their ethical sensitivity, ethical behavior during nursing practice can be standardized, reducing nurse-patient conflicts and facilitating the smoother integration of newly recruited nursing students into the hospital work environment.

*Perspective-taking plays a central role in the ethical sensitivity and empathy skills of nursing students*.

Nodes A5 (For use in patients at high risk of falls), A6 (Allowing dementia patients who are restrained to stay at the nurse station), and A7 (Performing bathing care after obtaining the consent of the opposite-sex patients) suggest that nursing students‘ ethical behavior during their initial internship is not solely guided by hospital regulations; rather, it is significantly influenced by their empathetic capabilities (44). This suggests that considering the patient's perspective and empathizing with their situation is a fundamental aspect of empathy, with emotional experience serving as the foundation of moral behavior (45). Perspective-taking allows nursing students to gain a deeper understanding of patients' physiological and psychological needs, which in turn facilitates prompt recognition of the patients‘ ethical rights and needs. This capability helps students detect potential violations of patient privacy or autonomy in their nursing practice (46). Empathy skills training can effectively enhance nursing students' empathetic abilities (47). However, the limitations of the current research context should be acknowledged. The distribution of teaching hours for nursing ethics courses, humanistic clinical training, and ethical scenario exercises related to restraint varies significantly across provincial colleges. Thus, the conclusions drawn from this study, based on the local college internship teaching model, cannot be universally applied to nursing students from diverse training systems nationwide. Consequently, empathy training and peer learning opportunities among students from different institutions should be encouraged (48). Future research could implement interventions designed to assess changes in empathy and ethical sensitivity among nursing students across various educational systems. The relationship between empathy and ethical sensitivity is critical; throughout nursing students‘ clinical practice, empathy activates their ethical behavior principles (49) and serves as a guiding force in their nursing actions and decisions. By putting themselves in others' shoes, nursing students can make choices that align with professional ethics while also embodying human compassion when confronted with ethical dilemmas such as those represented by A5, A6, and A7.

*The report's selection reflects the ethical responsibilities and role awareness of the nursing students*.

A13 (Reporting patient care details to the head nurse) stands out as the sole “communication-related” bridge node, highlighting the critical role of information transmission in linking empathy to ethical sensitivity. Comparative analysis with item A12 (Reporting to the responsible nurse) indicates significant differences in reporting levels between the two; A13 uniquely serves as the intermediary. At the onset of their internships, nursing students often lack a stable understanding of their roles (50). The decision to report patient conditions to the head nurse reflects a recognition of the head nurse's professional authority and management responsibilities. In nursing management, the head nurse acts not only as an executor of systems but also as a coordinator of doctor-nurse and nurse-patient relationships, as well as a controller of nursing quality (51). This communication behavior signifies nursing students' comprehension of the “hierarchical collaboration” inherent in the nursing management system (52). Contrary to A12, node A13 emphasizes that the reported content pertains to nursing details rather than the patient's general condition. Effective communication is essential in preventing nursing errors and minimizing nurse-patient disputes (53). By furnishing detailed reports, nursing students actively identify potential risks and seek professional guidance, thereby fulfilling their ethical obligation of “preventive communication.” Nursing ethics mandate that nurses demonstrate responsibility toward both their patients and the profession, with comprehensive reporting being a concrete expression of this responsibility. Empathy enhances the ability to recognize the potential repercussions of incomplete communication on the continuity of patient care (54), motivating students to adopt a more rigorous and responsible approach to reporting. Thus, it is essential that clinical nursing staff respect the responsibility awareness nursing students develop early in their education. Targeted, stratified, and standardized training in clinical communication should be implemented, along with a clear definition of nursing information reporting content (55). Such measures will not only ensure patient safety but also enhance the professional communication skills of nursing interns, facilitating the integration of empathy, ethical understanding, and clinical nursing practice.

## 5 Limitations

This study employs a cross-sectional research design, enabling the simultaneous collection of data on the ethical sensitivity and empathy ability of nursing students at the beginning of their internship. While this approach allows for the analysis of correlations between variables, it does not facilitate the establishment of temporal sequences or causal associations. Consequently, it fails to track the dynamic changes of these indicators throughout the entirety of the nursing students‘ internship experience. Additionally, the study utilized cluster sampling exclusively from a single tertiary hospital in Zibo City, leading to a single-center sample. This limited geographical scope introduces significant cultural specificity to the research conclusions, necessitating future studies to encompass multi-center and cross-regional research. Furthermore, all core variables were assessed through self-report scales completed by the nursing students, resulting in two types of measurement biases. The first is the common self-report bias, while the second relates to social expectation bias, whereby nursing students may respond in ways that align with social moral standards rather than truthfully reflecting their actual cognitions. The absence of objective behavioral observation indicators, such as clinical behavior assessments or third-party evaluations by teaching staff, means that the quantitative scores derived from the scales may not accurately capture the nursing students' real ethical behavior and empathetic performance in clinical settings. Finally, although the sample size meets the basic requirements for bridge network analysis and has undergone stability analysis confirming the overall network's acceptable robustness, certain key bridge nodes require further verification regarding their stability. Future research should aim to expand the sample size and conduct large-scale, multi-center surveys across the country to better assess the robustness of the bridge network structure and node effects.

## 6 Conclusion

This study focused on the ethical sensitivity and empathy ability of nursing students during the early stages of their internship. By utilizing network analysis methods, it systematically explored the characteristics of the connection network between these two dimensions and their interrelations. The analysis revealed that nodes A5, A6, A7, and A13 (Items 5, 6, 7, and 13 of the ethical sensitivity scale) and B1, B6, and B11 (Items 1, 6, and 11 of the empathy scale) served as bridging nodes mediating interactions between these dimensions, thereby illustrating the underlying mechanism of “empathy triggering moral behavior.” Based on these findings, the study emphasizes the need to enhance clinical teaching by concentrating on the cultivation of bridge links in ethical dilemmas. Through situational simulation and reflective teaching methodologies, both empathy ability and ethical awareness should be reinforced to prevent declines in empathy during the internship. This approach aims to foster nursing professionals who possess both substantial knowledge and strong humanistic qualities. A noted limitation is the study's focus solely on the initial internship stage, suggesting that future longitudinal research is needed to further investigate the dynamic characteristics of the bridge connection network.

## Data Availability

The original contributions presented in the study are included in the article/supplementary material, further inquiries can be directed to the corresponding author.

## Appendix

**Appendix 1:** Specific item names for each code.

Code	Item name
A1	Installing side rails around the bed to prevent patient falls
A2	A postoperative patient refuses to reposition due to pain, but repositioning is still required to prevent postoperative complications
A3	A terminally ill patient refuses to reposition due to respiratory discomfort from movement, but repositioning is still required every 2 hours due to high risk of pressure injuries
A4	Sending an elderly patient who wishes to return home to a nursing home, as the patient will have no family to take care of them after discharge
A5	If using sensors, they should be placed beside the bed of patients at high risk of falls
A6	Allowing a dementia patient who is sitting in a wheelchair and wearing a seatbelt to stay in the nurse's station
A7	A heterosexual patient refuses to receive care during bathing, but due to the patient's condition and safety needs, care should be provided after obtaining the patient's consent
A8	When administering medication to a dementia patient who refuses to take it, the medication may be mixed with the patient's drink without the patient's knowledge
A9	A terminally ill patient wishes to use the bathroom, and the nurse escorts the patient to the bathroom and provides assistance
A10	A bedridden patient who has long received bed baths wishes to take regular showers, and the nurse assists the patient with regular bathing
A11	Providing feeding assistance to a patient with dysphagia by adapting the eating speed, including continuous supervision for at least 1 hour
A12	Reporting the patient's condition to the charge nurse in the ward
A13	Reporting the detailed situation of the patient's care to the head nurse of the ward
B1	In my relationship with patients, understanding the emotional state of patients and their families is a very important factor
B2	For me, it is almost impossible to look at things from the patient's perspective
B3	Understanding the feelings of patients and their families is irrelevant to treatment
B4	Without empathy, I would find it difficult to become a successful nurse
B5	When I deeply empathize with my patients, they feel better
B6	In my relationship with patients, understanding their body language and verbal communication is equally important
B7	When observing a patient's condition and taking a medical history, I try not to pay attention to the patient's emotional changes
B8	I will pay attention to the patient's body language and non-verbal cues to understand what the patient is thinking
B9	I do not allow myself to be moved by the strong emotional relationship between the patient and their family
B10	I believe that empathy is an important factor in the treatment process
B11	To provide better nursing care, I will try to consider issues from the patient's perspective
B12	Diseases can only be treated with drugs or surgery, so establishing an emotional connection with the patient has no clear benefit for treatment
B13	When I deeply empathize with my patients, they feel that the treatment is effective
B14	Paying attention to the patient's personal experiences has no relationship with the treatment outcome
B15	I believe that asking patients about what happens in their daily lives does not help in understanding their condition
B16	I believe that a sense of humor helps patients receive better clinical treatment outcomes
B17	For me, it is very difficult to think from the patient's perspective
B18	I do not like reading books related to literature or art that are unrelated to medical care
B19	When caring for patients, I will try to think from the patient's standpoint
B20	I believe that emotional investment has no role in the treatment of diseases
